# Lifestyle management in polycystic ovary syndrome – beyond diet and physical activity

**DOI:** 10.1186/s12902-022-01208-y

**Published:** 2023-01-16

**Authors:** Stephanie Cowan, Siew Lim, Chelsea Alycia, Stephanie Pirotta, Rebecca Thomson, Melanie Gibson-Helm, Rebecca Blackmore, Negar Naderpoor, Christie Bennett, Carolyn Ee, Vibhuti Rao, Aya Mousa, Simon Alesi, Lisa Moran

**Affiliations:** 1 Monash Centre for Health Research and Implementation, Monash University, Clayton, Victoria Australia; 2 Eastern Health Clinical School, Monash University, Box Hill, Victoria Australia; 3 Health and Social Care Unit, Monash University, Clayton, Victoria Australia; 4 Robinson Research Institute, The University of Adelaide, North Adelaide, South Australia Australia; 5 Te Tātai Hauora o Hine – National Centre for Women’s Health Research Aotearoa, Te Herenga Waka - Victoria University of Wellington, Wellington, New Zealand; 6 Centre for Mental Health, Swinburne University of Technology, Hawthorn, Victoria Australia; 7 Department of Nutrition, Dietetics and Food, Monash University, Notting Hill, Victoria Australia; 8 NICM Health Research Institute, Western Sydney University, Westmead, New South Wales Australia

**Keywords:** Polycystic ovary syndrome, diet, guideline, physical activity, sleep, cognitive behavioural therapy, quality of life, complementary medicine

## Abstract

Polycystic ovary syndrome (PCOS) is a common condition affecting reproductive-aged women with reproductive, metabolic and psychological consequences. Weight and lifestyle (diet, physical activity and behavioural) management are first-line therapy in international evidence-based guidelines for PCOS. While these recommend following population-level diet and physical activity guidelines, there is ongoing interest and research in the potential benefit of including psychological and sleep interventions, as well as a range of traditional, complimentary and integrative medicine (TCIM) approaches, for optimal management of PCOS. There is limited evidence to recommend a specific diet composition for PCOS with approaches including modifying protein, carbohydrate or fat quality or quantity generally having similar effects on the presentations of PCOS. With regards to physical activity, promising evidence supports the provision of vigorous aerobic exercise, which has been shown to improve body composition, cardiorespiratory fitness and insulin resistance. Psychological and sleep interventions are also important considerations, with women displaying poor emotional wellbeing and higher rates of clinical and subclinical sleep disturbance, potentially limiting their ability to make positive lifestyle change. While optimising sleep and emotional wellbeing may aid symptom management in PCOS, research exploring the efficacy of clinical interventions is lacking. Uptake of TCIM approaches, in particular supplement and herbal medicine use, by women with PCOS is growing. However, there is currently insufficient evidence to support integration into routine clinical practice. Research investigating inositol supplementation have produced the most promising findings, showing improved metabolic profiles and reduced hyperandrogenism. Findings for other supplements, herbal medicines, acupuncture and yoga is so far inconsistent, and to reduce heterogeneity more research in specific PCOS populations, (e.g. defined age and BMI ranges) and consistent approaches to intervention delivery, duration and comparators are needed. While there are a range of lifestyle components in addition to population-recommendations for diet and physical activity of potential benefit in PCOS, robust clinical trials are warranted to expand the relatively limited evidence-base regarding holistic lifestyle management. With consumer interest in holistic healthcare rising, healthcare providers will be required to broaden their knowledge pertaining to how these therapies can be safely and appropriately utilised as adjuncts to conventional medical management.

## Introduction

Polycystic ovary syndrome (PCOS) is a common condition affecting up to 13% of reproductive-aged women [1]. It is diagnosed through the European Society for Human Reproduction and Embryology/American Society for Reproductive Medicine (ESRHE/ASRM) criteria, requiring two of the following features: polycystic ovaries on ultrasound, oligoovulatory or anovulatory cycles and biochemical or clinical hyperandrogenism [2]. Women with PCOS experience a combination of reproductive (infertility, pregnancy complications) [3], metabolic (risk factors for and conditions of type 2 diabetes (T2DM) and cardiovascular disease (CVD)) [4, 5] and psychological (conditions including anxiety, depression, poor quality of life (QoL), disordered eating) comorbidities [6, 7].

Insulin resistance (IR) is defined as a key pathophysiological feature in PCOS, contributing to hyperandrogenism and worsening the clinical presentation of PCOS. While lean women present with IR in a form that is mechanistically different from IR caused by excess weight, overweight and obesity further exacerbate IR and consequent hyperinsulinaemia [8]. Women with PCOS also display a higher rate of weight gain over time [9] and a greater prevalence of overweight and obesity [10], which can further contribute to this worsening of IR and hence worsening of the presentation of PCOS [11]. The reason for this is unclear, but may be related to differences in intrinsic psychological and biological mechanisms [12–15], or extrinsic lifestyle factors such as diet and physical activity [16, 17]. Improving IR and excess adiposity are therefore key targets in PCOS management.

The International Evidence-Based Guideline for the Assessment and Management of PCOS [18], highlights lifestyle intervention as the primary early management strategy. Lifestyle interventions are traditionally defined as those designed to improve dietary intake or physical activity through appropriate behavioural support. In the 2018 PCOS guideline, lifestyle management is recommended for general health benefits [18]. Given that excess weight is associated with increased IR in PCOS [8], the guideline additionally promotes weight management, defined as: 1) weight gain prevention in all women with PCOS, and 2) achieving and maintaining modest weight loss in women with excess weight [18].

Lifestyle interventions in PCOS management can also be viewed as a broader construct beyond physical health. Since the emergence of the biopsychosocial model of healthcare in 1977, health disciplines have seen a gradual shift away from the classical biomedical model (where health is defined as the ‘absence of disease’) towards whole person or holistic care [19]. This is an approach that reflects many facets of the patient context, via integrating care that addresses biological, psychological, social, spiritual and ecological aspects [20]. It therefore requires a range of different treatment strategies to improve health. Provision of whole person or holistic care has been identified as a core objective of healthcare reforms internationally [21–23]. In line with these reforms the PCOS guideline recognises the importance of emotional wellbeing to overall health and QoL in women living with PCOS [18]. It also highlights evidence which suggests that the psychological impact associated with PCOS is under-appreciated in clinical care [4, 5], and that few women are satisfied with the mental health support they receive [6, 7]. Recommendations for appropriate screening, assessment and treatment strategies for anxiety, depression, psychosexual dysfunction, eating disorders and poor body image are provided [18]. These specific areas of emotional wellbeing are of particular concern, with research showing a higher prevalence and severity of depression and anxiety [24, 25], lower scores for satisfaction with sex life and feeling sexually attractive [26] and a higher prevalence of disordered eating and eating disorders [7] in women with PCOS. Features of PCOS, in particular hirsutism and increased weight, have also been shown to negatively affect body image [27, 28], with poor body image being strongly related to depression in women with PCOS [29, 30].

**Fig. 1:**
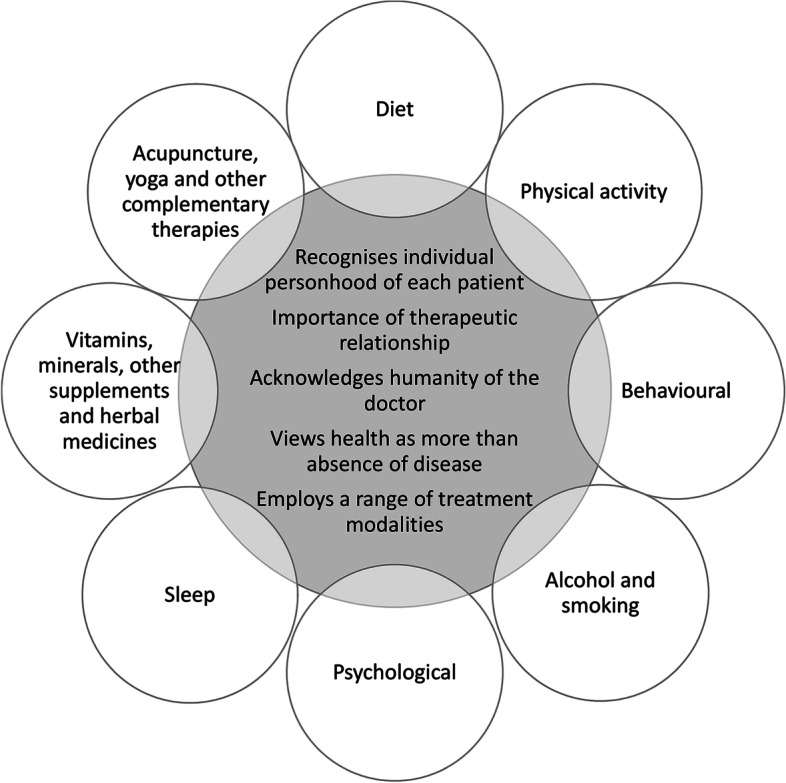
Viewing lifestyle modifications through a whole person or holistic care lens. The key features of whole person or holistic care listed in the centre of the figure have been adapted from Thomas et al. [20]. ‘Recognises individual personhood’ relates to focusing on the unique needs of the person rather than the disease. ‘Importance of therapeutic relationship’ emphasises patient autonomy and responsibility. ‘Acknowledges humanity of the doctor’ considers the doctors’ ability to self-reflect on how they engage in the care of the patient. ‘Health as more than absence of disease’ incorporates the mental, emotional, physical, environmental and social needs of the patient. ‘Employs a range of treatment modalities’ promotes continuity of care across health disciplines, and while it may include traditional, complementary and integrative medicine (TCIM), TCIM is not holistic if used in isolation and without adequate integration into conventional healthcare

While the current PCOS guideline is comprehensive, considering all available evidence at the time of development and providing best-practice recommendations for necessary screening, risk assessment and management, it could not possibly cover all aspects of PCOS care. An International Delphi process was used to prioritise clinical questions, with consensus reached through extensive consultation with both consumers and multidisciplinary clinicians with expertise in PCOS care. Therapies, such as traditional, complementary and integrative medicine (TCIM), supplement use, sleep and meditation interventions are either briefly considered or not at all included in the 2018 PCOS guideline. Many of these therapies are novel and there is a paucity of evidence to support intervention efficacy on PCOS outcomes. However, as patient interest in these types of non-pharmacological interventions are growing [31–35], it is prudent to provide more guidance to healthcare providers in this area on their potential efficacy in PCOS. Whole person or holistic care recognises that the doctor-patient relationship should be one of open dialogue, where healthcare providers involve the patient in negotiating their care and recognises patient’s autonomy to guide treatment (Figure 1) [36].

This review provides an extensive overview of evidence to date on lifestyle strategies used to optimise management of PCOS. Using a holistic definition of patient care, this review considers the traditional components of lifestyle change (diet, physical activity and behavioural change), psychological and sleep interventions, as well as TCIM approaches (supplements, herbal medicine, acupuncture and yoga). To improve translation of findings, evidence summaries are accompanied by an overview of relevant recommendations from the existing PCOS guideline. This highlights where emerging evidence supports current recommendations or provides new insights for research. As this is a narrative review, while evidence summaries include peer-reviewed journal articles identified from databases including Medline OVID, this is supplemented by expert opinion of the authors.

## Traditional lifestyle and weight management

The PCOS guideline recommends the promotion of healthy lifestyle behaviours in all women with PCOS, to achieve and/or maintain a healthy weight and to optimise general health [18]. In women with excess weight, a weight loss of 5-10% is advised, aiming for an energy deficit of 30% or 500-750 kcal/day (1200-1500 kcal/day). While weight management is seen as a core component of lifestyle interventions, the guideline recognises that a healthy lifestyle provides benefits that occur independent of weight change.

A recent Cochrane review of 15 randomised controlled trials (RCT) and 498 participants, reported that lifestyle interventions compared with minimal intervention or usual care, significantly reduces weight (kg) and body mass index (BMI) and improves secondary reproductive outcomes such as free androgen index (FAI), testosterone (T), sex hormone-binding globulin (SHBG) and hirsutism (Ferriman-Gallwey score) [37]. In terms of metabolic outcomes, lifestyle intervention resulted in significant reductions in total cholesterol (TC), low density lipoprotein cholesterol (LDL-C) and fasting insulin (FINS). These findings are largely similar to that of other systematic reviews [38–41]. While no studies in the Cochrane review assessed clinical reproductive outcomes [37], individual trials that were not included in the review have reported that lifestyle interventions resulting in modest weight loss (2-5% total body weight) improve ovulation and menstrual regularity [42–45]. Losing >5% of weight is additionally associated with being able to conceive, having live births, reduction of ovarian volume and reduction in the number of follicles [46–52].

Although weight loss has shown clear benefits to PCOS outcomes, including not only on reproductive function, but also glucoregulatory status, androgen status and lipid profiles [42–52], there are varying degrees of responsiveness to weight loss in terms of improvement of PCOS symptoms. One study by Pasquali et al. [53] found that when women achieved similar levels of weight loss (>5% weight) only one-third displayed a full recovery from PCOS, with the remainder showing only partial or no recovery. Higher waist circumference (WC), waist-hip-ratio (WHR) and androstenedione at baseline were associated with a poorer chance of successful outcomes [53], suggesting that central adiposity and more severe hyperandrogenism may predict responsiveness to weight loss interventions in PCOS. Huber-Bucholz et al. [45] also reported women who achieve greater reductions in central fat and insulin sensitivity show greater symptom improvement with weight loss. This suggests that lifestyle interventions which simultaneously reduce IR and improve body composition (namely fat distribution), may help to optimise outcomes in PCOS management independent of changes in weight status.

### Diet

The 2018 PCOS guideline recognises there is insufficient evidence to suggest that any specific dietary approaches provide greater benefits on health outcomes [18]. Dietary recommendations may take on a variety of balanced dietary strategies according to the individual’s lifestyle needs and preferences, as per general population recommendations [18]. This advice is based on a systematic review comparing different dietary compositions (e.g. low carbohydrate, low glycaemic index (GI) and glycaemic load (GL), high protein, monounsaturated fatty acid (MUFA) enriched and fat counting diets) to best manage PCOS, identifying minimal differences between diets on anthropometric outcomes, concluding weight loss improves the presentation of PCOS regardless of dietary composition [16, 54]. There is now an emerging body of evidence that suggests a range of dietary strategies may produce favourable effects on PCOS features that occur independent of weight loss. It is important that the emerging findings from these studies are thoroughly considered to support consumer and health professional interests. To summarise current evidence this review has grouped diets in terms of those that modify carbohydrates, protein and fat, as well as specific dietary patterns.

#### Carbohydrates

The use of altered carbohydrate composition remains the most researched dietary approach for PCOS management. Two systematic reviews published after guideline inception support altered carbohydrate intake to improve intermediate markers of PCOS [55, 56], finding that altering carbohydrate type, as opposed to content, is preferable to better manage PCOS [55]. RCTs [57–72] and pre-post intervention studies [73–80] demonstrate that following a low GI/GL diet for at least eight weeks significantly reduces WC [55, 73, 74] and BMI when compared to high GI/GL [56] or a regular diet [73–76], although levels of weight loss are generally comparable to other dietary compositions [59, 60, 72, 74]. These reductions are proposed to be a result of decreased hunger, which may reduce energy intake and make it easier to follow dietary recommendations in the long-term [78, 81–84]. Low GI/GL diets also improve insulin sensitivity and reproductive hormones (T, SHBG, FAI) compared to high carbohydrate [16, 55, 57, 79, 85] or control diets [56, 59, 73–76], contributing to improvements in reproductive function, specifically menstrual regularly [60, 79]. Lastly, low GI/GL diets can improve risk factors for T2DM and CVD, including glucose [86, 87], TC [55, 56, 59, 75, 77], LDL-C [55, 59, 75, 85], TAG [55, 59, 73] and HDL-C [75], when compared to a regular or high GI/GL diet. It must be noted that beneficial effects of low GI/GL diets may also be attributed to proportional increases in protein and/or fat loads.

#### Protein

In women with PCOS higher protein intakes may be superior at supressing androgen levels when compared to high carbohydrate diets. Postprandial research has shown that high protein meals can reduce insulin and dehydroepidiandrosteone stimulation compared to meals rich in glucose [88]. Research in the general population has also shown that reduced appetite and energy intakes from low GI/GL diets are related to increased protein intakes [89, 90]. RCTs and pre-post intervention studies found that high protein diets (defined here as protein constituting ≥25% energy [91]) consumed for at least four weeks reduce weight [12, 73, 74, 92–96], BMI [73, 74, 92, 95], WC [73, 74, 92, 97], WHR [73] and fat mass [74, 92, 97]. These reductions in anthropometric measures are accompanied by improved FINS [12, 74, 95, 98] and HOMA-IR [12, 73, 95, 98], blood lipids [12, 96], T [73, 92, 94] and hirsutism (Ferriman-Gallwey score) [73]. However, only three of these studies were able to show significant improvements in anthropometric measures [97], insulin sensitivity [98] and blood lipids [12] when compared to low/standard protein [12, 97] or control diets [98]. Only one study investigated effects on mental health outcomes and found that a high protein diet reduced depression and improved self-esteem [99].

#### Fats

Fatty acid composition is also an important consideration as metabolic disorders associated with PCOS can benefit from increased MUFA and polyunsaturated fatty acid (PUFA) intakes [63–65]. Postprandial research in PCOS reported prolonged reductions in T for high fat compared to low fat meals, which likely results from delayed nutrient absorption [86]. Two acute meal studies in lean and obese women with and without PCOS reported that proatherogenic inflammatory markers [100] and oxidative stress [101] were elevated, independent of but augmented by obesity, following saturated fat ingestion with this associated with worsened IR and androgens. Two experimental studies in PCOS investigated the effects of habitual walnut (PUFA rich diet) [102, 103] and almond (MUFA rich diet) [102] intake for at least six weeks and reported no differences in glucoregulatory status, lipids or androgens with the exception of HbA1c significantly decreasing in the walnut relative to the almond group. Kasim-Karakas et al. [103] reported increased fasting and postprandial glucose (oral glucose tolerance test (OGTT)) for increased walnut intake compared to habitual (control), which they postulated may be related to the control diet being rich in oleic acid. Together these findings suggest minimal benefit for improving dietary PUFA compared to MUFA content. Two RCTs in women with PCOS investigated the effects of diets rich in olive [104, 105], canola [105] and sunflower [105] oil. Yahay et al. [105] reported 25g/day canola oil caused reductions in TAG, TC/HDL-C, LDL-C/HDL-C, TAG/HDL-C and HOMA, but not androgens, compared to 25 g/day olive and sunflower oils [105]. This may be related to the more favourable fatty acid composition of canola oil, with comparable MUFA content to olive oil, higher alpha-linolenic acid, lower omega-6/omega-3 ratio and saturated fat than both olive and sunflower oils. Douglas et al. [104] reported weight and the acute insulin response (OGTT) were lower following a eucaloric low carbohydrate compared to a eucaloric MUFA-enriched olive oil diet, suggesting that reduced carbohydrate intake may have grater glucoregulatory benefits than increased MUFA intake [104]. Lastly, two RCTs compared hypocaloric low-fat diets to a low carbohydrate [106] or low GI [107] diets, with reductions in weight [106], WC [106], body fat [106, 107], FINS [106] and FAI [106] in both groups but no difference between groups.

#### Dietary and eating patterns

In addition to diets that focus on specific macronutrient manipulations, there are a range of dietary patterns which have been explored in PCOS management. A systematic review (including 19 studies and 1,193 participants) published after guideline development (2020) found that the Dietary Approaches to Stop Hypertension (DASH) diet (rich in fruit, vegetables, wholegrains, nuts, legumes and low-fat dairy and with a predominantly low-GI carbohydrate profile) was the optimal choice for reducing IR [85]. RCTs in PCOS also report beneficial effects on weight [63, 64], BMI [62, 63], IR [62] and hormonal profile, including SHBG [64], androstenedione [64] and FAI [62] for a DASH compared to a control diet after 8-12 weeks. A vegetarian diet also reduced inflammatory markers (CRP, resistin and adiponectin) compared to a meat inclusive diet [80]. A vegan diet improved weight loss at three, but not six months [68], and a pulse-based diet led to similar reductions in weight, insulin sensitivity and reproductive hormones compared to a healthy control diet [72]. All of these dietary patterns are high in fibre and plant proteins, producing favourable effects on microbial diversity and encouraging production of short-chain fatty acids that possess potential anti-inflammatory actions [108, 109]. With mechanistic animal studies suggesting a possible pathophysiological role of gut microbiota in IR and ovarian dysfunction, it is possible that metabolic and hormonal benefits associated with plant-based dietary patterns in PCOS are related to increased intakes of dietary prebiotics [110]. However, further mechanistic studies exploring the role of gut microbiota in PCOS and RCTs investigating effects of dietary prebiotics on PCOS outcomes are required.

Lastly, particular eating patterns, such as eating smaller more frequent meals across the day [111] and eating a larger breakfast and smaller dinner [66], have also been found to be beneficial for insulin sensitivity [66, 111] and androgen reductions [66]. This is an important finding, as women with PCOS are more likely to either skip breakfast or consume their breakfast and lunch later in the day [112].

Studies examining specific food items in relation to PCOS outcomes, including raw onions [65], concentrated pomegranate juice [69, 113–115] and flaxseed powder [70, 116] have yielded largely inconsistent results. A core limitation of these single food studies is that foods are never consumed alone within the diet, omitting the influence of the dietary matrix and the interactions that occur amongst dietary constituents within meals. These studies provide limited applicability in the context of formulating practical dietary recommendations [117]. Please see Table 1 for a summary of available evidence from reviews and experimental studies investigating the effects of different types of diets on PCOS outcomes.

**Table 1:** Reviews and experimental studies investigating the effects of diet on polycystic ovary syndrome outcomes

Dietary intervention	N study design	N studies; N participants	Main findings^a^	References
Low CHO	2 SR/MA (27 RCT total - 18 RCT using low CHO diet) [56, 85] 1 SR (5 RCT total - 1 RCT using low CHO diet) [16]	14; 901	Low CHO compared with control diets [56]: ↓ BMI, HOMA-IR, TC, LDL-C ↑ SHBG ↔ LH, T, HDL-C Dietary intervention ↓HOMA-IR, FINS, FGL, BMI, BW, WC compared with minimal intervention, and subgroup analysis showed no additional benefits for low CHO diets [85] Weight loss improved the presentation of PCOS regardless of dietary composition, with no subtle differences observed for low CHO diets [16]	Shang et al. 2020 [85] Zhang et al. 2019 [56] Moran et al. 2013 [16]
Low GI	1 SR/MA (10 RCT total - 8 RCT using low GI diet) [55] 1 SR (5 RCT total - 1 RCT using low GI diet) [16] 4 Pre-post prospective [61, 76, 77, 118] 1 RCT [78]	16^b^; 582	High GI compared with low GI diets [55]: ↓ HOMA-IR, FINS, TC, LDL-C, TAG, WC, T ↔ FGL, HDL-C, BW, FAI Low GI diets had greater improvements in IR, TC, HDL-C, fibrinogen, menstrual regularity and QoL [16] Low GI diets followed for ≥ 12 weeks: ↓ BW [76, 77], BMI [76, 77], BFM [77], WC [77], WHR [77], FINS [76, 77], FGL [77], TC [77], LDL-C [77], TAG [77], T [77], LH [77], androstenedione [77], prolactin [77] ↑ insulin sensitivity (HOMA2-IS) [61], synthesis of predominantly anti-inflammatory eicosanoid mediators (e.g. 16(R)/16(S)-HETE, 13(S)-HODE, 9(S)-HODE, 15(S)-HETE, 12(S)-HETE, 5(S)-oxoETE, 5(S)-HETE)) [118], fasting glucagon (higher glucagon levels associated with lower levels of self-reported hunger) [78]	Kazemi et al. 2020 [55] Moran et al. 2013 [16] Shishrehgar et al. 2019 [76] Barr et al. 2016 [61] Szczuko et al. 2018 [77] Szczuko et al. 2017 [118] Hoover et al. 2021 [78]
High protein	1 SR (5 RCT total - 3 RCT using high protein diet) [16] 2 Pre-post prospective [73, 74] 6 RCT [92, 94–97]	11; 308	High protein diets improve depression and self-esteem [16] ↓ BFM [74, 97], BW [73, 74, 97], BMI [73, 74], WC [73, 74, 97], WHR [73], FINS [74, 98], FGL [97], HOMA-IR [73, 98], TAG [73], VLDL-C [73], T [73, 98], Ferriman-Gallway scores [73] High protein and standard/low protein diet ↓ BW [92, 94, 95], BMI [92, 94, 95], BFM [95], WC [92, 94], WHR [94], FINS [95, 96], HOMA-IR [95], TAG [96], LDL-C [98] CRP [96], MPA [96], leptin [95], T [98], DHEAS [98], FAI [98] and there was ↔ between high and standard/low protein diets	Moran et al. 2013 [16] Moran et al. 2010 [96] Moran et al. 2004 [95] Sorensenet et al. 2012 [97] Toscani et al. 2011 [92] Nadjarzadeh et al. 2021 [94] Phy et al. 2015 [73] Pohlmeier et al. 2014 [74] Mehrabani et al. 2012 [98]
Low fat	1 SR/MA (19 RCT total - 1 RCT using low fat diet) [85] 1 SR (5 RCT total - 1 RCT using low fat diet) [16] 1 RCT [107]	3; 137	Dietary intervention ↓HOMA-IR, FINS, FGL, BMI, BW, WC compared with minimal intervention, and subgroup analysis showed no additional benefits for low fat diets [85] Weight loss improved the presentation of PCOS regardless of dietary composition, with no subtle differences observed for low fat diets [16] Low fat (25% E fat) ↓ BW, BFM, BMI though there was ↔ between low fat and standard fat (35% E fat) diets [107]	Shang et al. 2020 [85] Moran et al. 2013 [16] Wong et al. 2016 [107]
Fatty acids	1 SR (5 RCT total - 1 RCT using MUFA enriched diet) [16] 3 RCT [86, 102, 105] 1 controlled trial (not randomised) [103]^c^	5; 146	MUFA enriched diets may produce greater weight loss when compared to other dietary patterns [16] MUFA enriched compared with PUFA enriched diets ↓ FGL [103], glucose response to OGTT [103], HgBA1 [102] Diets with a higher alpha-linolenic acid, lower omega-6/omega-3 ratio and saturated fat content ↓ TAG, TC/HDL-C, LDL-C-/HDL-C, TAG/HDL-C, and HOMA-IR [105] High total and saturated fat meals compared with high fibre low fat meals produce prolonged ↓ in T [86]	Moran et al. 2013 [16] Yahay et al. 2021 [105] Kalgaonkar et al. 2011 [102] Kasim-Karakas et al. 2004 [103] Katcher et al. 2009 [86]
DASH	1 SR/MA (19 RCT total - 4 RCT using DASH diet) [85]	4; 228	Dietary intervention ↓ HOMA-IR, FINS, FGL, BMI, BW, WC compared with minimal intervention, and subgroup analysis showed DASH diet was more effective at improving insulin sensitivity [85]	Shang et al. 2020 [85]
Plant-based	3 RCT	3; 108	Plant-based (35% animal protein, 35% textured soy protein, 30% vegetable protein) compared to control (70% animal protein, 30% vegetable protein) ↓ BMI, FGL, FINS, TAG, HOMA-IR, T, MDA and ↑ QUICKI [67] Plant-based and control diets (calorie restriction [68] and general dietary recommendations [72]) ↓ BW [68], HOMA-IR [72], T [72], LH/FSH [72] and there was ↔ between plant-based and control diets	Turner-McGrievy et al. 2014 [68] Kazemi et al. 2020 [72] Karamali et al. 2018 [67]
Meal pattern	1 RCT	1; 40	6 meals/day compared with 3 meals/day: ↓ FINS ↑ post-OGTT insulin sensitivity	Papakonstantinou et al. 2016 [111]
Meal timing	1 RCT	1; 60	Consuming a high kilojoule breakfast compared with a high kilojoule dinner: ↓ FGL, FINS, HOMA-IR, T ↑ SHBG	Jakubowicz et al. 2013 [66]

*Abbreviations*: ↑ significant increase (*P* ≤ 0.05), ↓ significant decrease (*P* ≤ 0.05), ↔ no significant change, *BFM* Body fat mass, *BMI* Body mass index, *BW* Body weight, *CHO* Carbohydrate, *CRP* C-reactive protein, *DHEAS* Dehydroepiandrosterone-sulfate, *E* Energy, *FAI* Free androgen index, *FBM* Fat body mass, *FGL* Fasting glucose level, *FINS* Fasting insulin level, *FSH* Follicle stimulating hormone, *GI* Glycaemic index, *HDL-C* High density lipoprotein cholesterol, *HOMA-B* Homeostatic Model Assessment for Beta Cells, *HOMA-IR* Homeostatic Model Assessment for Insulin Resistance, *IR* Insulin resistance, *LDL-C* Low density lipoprotein cholesterol, *LH* Luteinizing hormone, *MDA* Malondialdehyde, *MUFA* Monounsaturated fatty acid, *OGTT* Oral glucose tolerance test, *PCOS* Polycystic ovary syndrome, *PUFA* Polyunsaturated fatty acid, *QoL* Quality of life, *QUICKI* Quantitative insulin sensitivity check index, *SHBG* Sex hormone-binding globulin, *T* Testosterone, *TAG* Triglycerides, *TC* Total cholesterol, *VLDL-C* Very low density lipoprotein cholesterol, *WC* Waist circumference, *WHR* Waist hip ratio

^a^Summarises commonly used measures in PCOS research and does not report on all measured outcomes. For prospective pre-post studies significant changes from baseline are reported. For RCTs significant changes between intervention(s) and control are reported. Only experimental studies not already summarised in included systematic reviews/meta-analysis are presented

^b^Shishregar et al. 2019 total study population included 62 women though only findings for the women with PCOS (*n*=28) are included

^c^While habitual diet (control) was not enriched with MUFA, nutritional analysis showed that it was rich in oleic acid

### Physical activity

The 2018 PCOS guideline recommends ≥150 minutes per week of moderate or ≥75 minutes per week of vigorous intensity exercise for weight gain prevention, and ≥250 minutes per week of moderate or ≥150 minutes per week of vigorous intensity exercise for weight loss and weight regain prevention [18]. Minimising sedentary time and the inclusion of strength training exercise for two days per week is also recommended [18].

To date the most comprehensive review in PCOS (including 27 papers from 18 trials up until June 2017) reported that exercise improved FINS, HOMA-IR, TC, LDL-C, TAG, body composition (body fat percentage and WC) and aerobic fitness (VO_2max_) [119] compared with usual care or control groups. In regards to exercise type, subgroup analysis reported aerobic exercise improved BMI, WC, body fat percentage, FINS, HOMA-IR, TC, TAG and VO_2max_. In contrast, while resistance training produced unfavourable effects on HDL-C (decrease) and BMI (increase), it improved other measures of anthropometry, including WC. Combined interventions (using both aerobic and resistance training) had no effect on any of the measured markers. Subgroup analysis also found that more outcomes improved when interventions were supervised, of a shorter duration (≤ 12 weeks) and were conducted in women who were above a healthy weight [119].

Three more recent systematic reviews have looked at the effects of specific types of exercise on PCOS outcomes [120–122]. These reviews found that vigorous aerobic exercise can improve measures of insulin responsiveness and resistance, including HOMA-IR [121] and the insulin sensitivity index [120]; body composition, including WC [121] and BMI [122]; and cardiorespiratory fitness (VO_2max_) [121]. High intensity interval training (HIIT) alone may be effective for improving IR and BMI [123], however this has not been consistently shown [124]. Interventions involving a combination of aerobic and resistance exercise [122] or resistance training only [120] did not result in improvements in BMI [122] or weight status [120]. Exercise involving resistance training did result in other beneficial improvements to body composition (reduced body fat, WC and increased lean mass) and strength. This is important, as the degree of central adiposity predicts responsiveness to weight loss interventions in PCOS [53], and women who achieve greater reductions in central fat show greater symptom improvement with weight loss [45]. Resistance training may also improve androgen levels, though findings are inconsistent and more research is needed to draw definite conclusions [120]. There was insufficient evidence from available data to assess the effects of exercise type on reproductive function [122]. Please see Table 2 for a summary of available evidence from meta-analyses investigating the effects of different types of exercise on PCOS outcomes.

**Table 2:** Meta-analyses investigating the effects of different types of exercise on polycystic ovary syndrome outcomes

Physical activity intervention	N reviews; N studies; N participants	Main findings^a^	References
Aerobic exercise	4; 48; 1518	↓ WC [119, 121, 124], BMI [119, 122, 124], BF% [119], HOMA-IR [119, 121, 122, 124], TC [119, 124], FINS [119, 124], TAG [119], LDL-C [119], RHR [119] ↑ VO_2peak/max_ [119, 121, 124] ↔ BMI [121], BW [119, 124], HDL-C [124], LDL-C [124], TAG [124], FGL [119, 124], BP [119], HOMA-IR [122], FAI [119, 121, 122], T [119, 122], SHBG [119], E2 [119], LH [119, 122], FSH [119, 122]	Patten et al. 2020 [121] dos Santos et al. 2020 [122] Richards et al. 2021 [124] Kite et al. 2019 [119]
Resistance training	2; 14; 505	↓ WC [119], HOMA-IR [121], FINS [119], HDL-C [119], FAI [121] ↑ BMI [119] ↔ BW [119], BF% [119], FGL [119], HOMA-IR [119], TAG [119], TC [119], LDL-C [119], VO_2max/peak_ [119], RHR [119], FAI [119], T [119], SHBG [119], E2 [119], LH [119], FSH [119]	Patten et al. 2020 [121] Kite et al. 2019 [119]
Combined aerobic and resistance training^b^	2; 3; 59	↔ BMI [119, 122], WC [119], HOMA-IR [119, 122], FINS [119], FGL [119], BP [119], TAG [119], TC [119], LDL-C [119], HDL-C [119], RHR [119], T [119, 122], E2 [119], LH [119], FSH [119]	Kite et al. 2019 [119] dos Santos et al. 2020 [122]
High intensity interval training	2; 11; 373	↓ BMI [123], WHR [123], HOMA-IR [123, 124] ↔ BF% [123], BMI [124], BW [124], WC [124], TC [123, 124], LDL-C [123, 124], TAG [124], FINS [123, 124], FGL [124], HDL-C [124], VO_2max_ [124]	Richards et al. 2021 [124] dos Santos et al. 2021 [123]

*Abbreviations*: ↑ significant increase (*P* ≤ 0.05), ↓ significant decrease (*P* ≤ 0.05), ↔ no significant change, *BF%* Percent body fat, *BMI* Body mass index, *BW* Body weight, *BP* Blood pressure, *E2* Estradiol, *FGL* Fasting glucose level, *FAI* Free androgen index, *FINS* Fasting insulin, *FSH* Follicle stimulating hormone, *HOMA-IR* Homeostatic assessment of insulin resistance, *LDL-C* Low density lipoprotein cholesterol, *LH* Luteinizing hormone, *PCOS* Polycystic ovary syndrome, *RHR* Resting heart rate, *SHBG* Sex hormone-binding globulin, *T* Testosterone, *TAG* Triglycerides, *TC* Total cholesterol, *VO2max* Maximal oxygen uptake, *VO2peak* Peak oxygen uptake, *WC* Waist circumference, *WHR* Waist hip ratio

^a^Significant findings from meta-analyses when comparing exercise groups to control

^b^Subgroup analyses compared different types of exercise; only 1 study included for combined exercise

When comparing the effects of exercise and diet combined with diet alone, a systematic review and meta-analysis (three studies) found no differences for any measured outcomes (glucose, insulin HOMA-IR, weight, BMI, WC, body fat, fat free mass, T, SHBG and FAI) [119]. In regards to exercise and diet combined compared to exercise alone, subgroup analysis (including 17 studies) from a large systematic review found that the addition of diet to exercise, particularly vigorous intensity aerobic exercise, resulted in greater reduction to BMI, WC, FAI and HOMA-IR than exercise only [121]. In regards to exercise (aerobic) alone versus diet alone, one intervention study found that exercise induced weight loss produced greater improvements in menstrual frequency and ovulation rates [125], with no differences in pregnancy rates [125]. However, this study was not randomised and treatments were self-selected, which may have biased the results and precludes firm conclusions [125].

### Behavioural

The 2018 PCOS guideline promotes the use of behavioural interventions that foster self-efficacy [18]. These include the use of SMART (specific, measurement, achievable, realistic and timely) goals, self-monitoring, stimulus control, problem solving and relapse prevention [18].

Behavioural and cognitive interventions are required to improve sustainability of lifestyle changes, through considering not only the specific behaviour, but also their antecedents, consequences and cognition [126, 127]. Given that women with PCOS show higher rates of weight gain over time [9] and high attrition rates in clinical weight management research [37], there is a clear need to improve adherence to diet and physical activity interventions. However, the majority of research investigating lifestyle change in PCOS involve short-term dietary interventions with/without an exercise element, and there is a paucity of research on behavioural change strategies. As such, guideline development relied heavily on evidence taken from the general population. Only three RCTs in women with PCOS included a ‘behavioural intervention’ [128–130]. While these studies showed enhanced weight loss [128, 130] and improved androgen and lipid profiles [129] when compared with placebo, the interventions were not well defined, with negligible context provided regarding the theoretic framework or behavioural strategies utilised.

More recently, a cross-sectional study in 501 women with PCOS [131] and two RCTs [44, 132] explored the use of self-management strategies [131] and behavioural modification interventions [44, 132] in PCOS. In the cross-sectional study, implementation of physical activity self-management strategies improved the likelihood of meeting physical activity recommendations, but had no association with BMI. Dietary self-management strategies were associated with reductions in BMI, though were not related to weight or nutritional intake [131]. In the RCTs, only the behavioural modification programme and not the control (general healthy lifestyle recommendations) produced significant weight loss after four months. A significantly greater proportion of women in the intervention group also improved menstrual regularity [44] and psychological well-being (lower anxiety and depressive symptoms) [132] when compared to the control group. The women who achieved greater weight loss reported higher social desirability and lower embitterment scores on a personality trait assessment measure [132]. These findings are particularly novel, as they provide insight into the influence of personality traits and their contribution to success in following behavioural modifications [132].

### Alcohol and smoking

In the clinical setting, smoking and alcohol consumption are often addressed alongside dietary and physical activity changes, employing the same behavioural and cognitive interventions to promote adherence. Hence, alcohol and cigarette use are considered here under traditional lifestyle strategies. The PCOS international guideline highlights the importance of assessing alcohol consumption and cigarette smoking when improving fertility and reproductive outcomes in women with PCOS [18]. Assessment of cigarette use is also recommended when evaluating CVD risk factors and thromboembolism risk associated with oral contraceptive pills [18]. These recommendations are based on existing practice guidelines used for the general population.

There is a paucity of observational research characterising alcohol consumption in women with PCOS. One Swedish study comparing women with PCOS (*n*=72) to healthy controls (*n*=30), demonstrated a lower alcohol intake in the PCOS group [133]. A larger study in Australia comparing women with (*n*=409) and without (*n*=7,057) PCOS, reported no significant difference in alcohol intake [134]. Similarly, a Spanish study (*n*=22 PCOS and *n*=59 controls) and a Chinese study (*n*=2,217 PCOS and *n*=279 controls), found no significant difference in alcohol intake between PCOS and non-PCOS groups [135, 136].

Current evidence on the impact of alcohol intake on anovulatory infertility (a common feature of PCOS) is controversial, with some studies showing adverse effects and others reporting no significant correlation [136, 137]. One prospective study including 18,555 married women from The Nurses’ Health Study II, who had no history of infertility, found no clinically significant impact of alcohol intake on anovulatory infertility, after adjusting for parity and other factors [138]. Similarly, a Danish study (*n*=6,120 women aged 21 to 45 years) found no fertility effect with alcohol consumption of less than 14 standard drinks per week [137]. In contrast, a study on 3,833 women who recently gave birth and 1,050 women with infertility, reported an increased risk of anovulatory infertility and endometriosis with increasing alcohol intake [139].

Current observational evidence does not reveal any significant difference in smoking between women with and without PCOS [135, 136, 140], with the exception of one study in pregnant women which showed a lower smoking rate in women with PCOS (*n*=354) compared to women without PCOS at 15 weeks gestation [3]. However, a significantly higher rate of smoking (including passive and active) is reported in women with PCOS and oligo-anovulation and/or reduced fertility compared to women with PCOS and normal menstruations or healthy controls [141, 142]. Smoking is also associated with PCOS risk independent of BMI and age [142]. A Mendelian randomisation study supports these findings, demonstrating a 38% higher risk of PCOS development in genetically predicted smokers (based on single-nucleotide polymorphisms associated with smoking initiation) compared with those who never smoked [143]. In PCOS, smoking is associated with increased levels of T, DHEAS, TC, LDL-C and FINS [141, 144, 145]. However, the underlying mechanisms are not fully understood and there are inconsistencies in findings from different studies. Furthermore, smoking is associated with lower conception and live birth rates and less favourable ART outcomes in women with PCOS [141, 146].

## Psychological

The current guideline highlights the need for awareness, and appropriate assessment (such as stepwise screening) and management, of QoL, depression and anxiety, psychosexual dysfunction, negative body image and disordered eating [18]. The guideline emphasises the importance of clinicians and women working in partnership to address women’s individual priorities; understanding that the impact of PCOS on an individual’s QoL is key to delivering meaningful outcomes [147, 148]. To assist women to communicate with clinicians about what is important to them, the PCOS Question Prompt List [149] was developed and is consistent with the 2018 guideline. The 2018 guideline recommends screening for risk factors and symptoms of depression and anxiety at time of diagnosis. Women with positive screening results should be supported with further assessment and treatment by appropriately qualified clinicians. To screen for psychosexual dysfunction tools such as the Female Sexual Function Index [150] should be utilised. If negative body image, disordered eating or eating disorders are suspected, the PCOS guideline outlines a stepped approach for screening, and where appropriate promotes the use of psychological therapy offered by trained health professionals, which should be guided by regional clinical practice guidelines [18].

While the PCOS guideline provides justification and summarises evidence for mental health screening and diagnostic assessment, there is also a need for consideration of additional aspects, such as the efficacy of different types of psychological interventions and how psychological interventions influence engagement with lifestyle change. This is important, as poorer mental health outcomes at baseline are positively associated with higher rates of attrition in lifestyle interventions [13]. Cognitive behavioural interventions could be considered to improve engagement and adherence to healthy lifestyle in women with PCOS. Research has shown support for a range of different psychological interventions, such as counselling [151], cognitive behavioural therapy (CBT) [152–154] and mindfulness meditation [155, 156], helping to change the way clinicians’ approach and deliver optimal PCOS management.

CBT is one of the most widely-researched psychological interventions, and is well-recognised as the most effective psychological treatment for depression and anxiety [157]. One RCT showed that eight weekly group CBT sessions were effective in improving QoL ratings and reducing psychological fatigue in women with PCOS [152]. Another more recent RCT investigated the outcome of a 1 year three-component intervention focusing on CBT, diet and exercise [154] and reported improvements in self-esteem and depressive symptoms as compared to usual care [154]. Similarly, an RCT by Cooney et al. [153], comparing the effects of CBT and lifestyle modification versus lifestyle modification alone, reported the CBT/lifestyle modification group lost more than twice as much weight per week and had greater improvements in QoL compared to lifestyle only. Depression scores decreased in the overall group and there was no difference between the two groups [153]. Lastly, a pilot intervention study of adolescents with PCOS has shown promising results for the use of CBT in the reduction of weight and improvement in depressive symptoms [158].

Mindfulness meditation programs have gained increasing popularity over the past few decades, and are being included as part of clinical trials to reduce stress and improve psychological wellbeing across a range of medical conditions [159]. Mindfulness meditation can be used to reduce the production of adrenal androgens, activated via the adrenal glands as a direct result of psychological distress [156]. Despite the proposed benefits, there are very few studies investigating the use of mindfulness meditation as a treatment for psychological symptoms associated with PCOS. One RCT (*n*=86) compared the provision of an eight week mindfulness-based stress reduction (MBSR) program, and found that when compared to the control group (health education), the MBSR group produced greater reductions in perceived stress, depressive symptoms and fasting blood glucose [160]. Similarly, another RCT investigating the impact of mindfulness meditation for eight weeks in PCOS showed reduced stress, depression and anxiety symptoms, and increased life satisfaction and QoL in the intervention group compared to no treatment [156]. In adolescents with PCOS (*n*=37), a pilot RCT reported higher levels of nutrition and physical activity self-efficacy following a mindfulness and self-management program [161]. Mindfulness-based cognitive therapy (MBCT) combines both elements of MBSR and CBT, but as yet there are no trials investigating this intervention in PCOS.

In addition to CBT and mindfulness meditation, there is some evidence to support group counselling sessions as beneficial in conjunction with exercise programs to increase and support weight loss [151]. In one RCT (*n*=17) participants followed a high-intensity aerobic exercise program for eight weeks, followed by eight weeks of group counselling [151]. Qualitative analysis of data taken from the group counselling and physical exercise sessions revealed that development of supportive relationships was important for successful behavioural change. By fostering the exchange of narratives relating to their illness (e.g. effects of PCOS on aspects of everyday life), and generating feedback between group members, counselling sessions helped to reduce social isolation and improve adherence to the exercise intervention [151]. Please see Table 3 for a summary of experimental studies investigating effects of psychological interventions on PCOS outcomes.

**Table 3:** Experimental studies investigating the effects of psychological interventions on polycystic ovary syndrome outcomes

References	Study design; study length; N participants	Intervention	Main findings
Abdollahi et al. 2019 [152]	Parallel RCT; 8 wk; 74	I = 8 weekly CBT C = minimal intervention	↑ QoL (PCOSQ) for I compared with C ↓ psychological fatigue (FIS) for I compared with C
Jiskoot et al. 2020 [162] Jiskoot et al. 2020 [154]	Parallel RCT; 1 yr; 183	I = 20 group sessions of CBT combined with nutrition advice and exercise C = usual care	↓ depression (BDI-II) and BW in I compared with C ↑ self-esteem (RSES) in I compared with C
Oberg et al. 2020 [132]	Parallel RCT; 16 wk with a follow-up at 1 yr; 68	I = behavioural modification program C = minimal intervention	↓ anxiety (PGWBI) and depressed mood (PGWBI) in I compared with C ↑ higher general health (PGWBI) in I compared with C
Cooney et al. 2018 [153]	Parallel RCT; 16 wk; 31	I = 8 weekly CBT with lifestyle modification C = no psychological intervention with lifestyle modification	↓ BW in I compared with control ↑ QoL (PCOSQ) in I compared with control
Raja-Khan et al. 2017 [160]	Parallel RCT; 16 wk; 86	I = 8 weekly MBSR C = 8 weekly health education sessions (diet and exercise education)	↑ mindfulness (TMS) in I compared with C ↓ perceived stress (PSS-10) in I compared with C
Stefanaki et al. 2015 [156]	Parallel RCT; 8 wk; 38	I = MBSR C = minimal intervention	↓ depression (DASS21), stress (DASS21) and cortisol in I compared with control
Roessler et al. 2012 [151]^a^ Roessler et al. 2013 [163]^b^	Cross-over randomised; 8 wk per arm and 16 wk total; 17	8 wk high-intensity aerobic exercise (including a ramp-up period of two weeks) and 8 wk group counselling in a cross-over design without a wash-out period	Relationships between the participants were important for changes in behaviour, especially relationships which generated helpful peer feedback and reduced social isolation ↓ BW and BMI after 16 wk only in the group who started with group counselling
Rofey et al. 2009 [158]	Single arm experimental; 8 wk; 12	8 one-on-one CBT, 3 family-based CBT and lifestyle goals (diet and exercise)	↓ BW, BMI and depression (CDI) ↑ health-related QoL (IWQoL-K)

*Abbreviations*: ↑ significant increase (*P* ≤ 0.05), ↓ significant decrease (*P* ≤ 0.05), *BDI-II* Beck Depression Inventory-II, *BP* Blood pressure, *BMI* Body mass index, *BW* Body weight, *CDI* Children’s Depression Inventory, C Control, *CBT* Cognitive behavioural therapy, *CES-D* Centre for Epidemiologic Studies – Depression Scale, *DASS21* Depression Anxiety Stress Scales-21, *DSM-IV* Diagnostic and Statistical Manual of Mental Disorders (fourth edition), *FGL* Fasting glucose level, *FIS* Fatigue Impact Scale, *I* Intervention, *IWQoL-K* Impact of Weight on Quality of Life Questionnaire—Kids, *HP* Hip circumference, *MBSR* Mindfulness-based stress reduction, *PSS-10* Perceived Stress Scale-10, *PCOS* Polycystic ovary syndrome, *PCOSQ* Polycystic Ovary Syndrome Health-Related Quality of Life Questionnaire, *PGWBI* Psychological Well-Being Index, *QoL* Quality of life, *RSES* Rosenberg Self Esteem Scale, *RCT* Randomized controlled trial, *STAI* State-Trait Anxiety Inventory, *SSP* Swedish Universities Scale of Personalities, *TMS* Toronto Mindfulness Scale, *TSST* Trier Social Stress Test, *WC* Waist circumference

^a^Qualitative analysis only

^b^Statistical analysis compares order of intervention arms (e.g. counselling followed by exercise versus exercise followed by counselling) and doesn’t compare effects of counselling versus exercise

## Sleep

Women with PCOS have an increased risk of both clinical sleep disorders and non-clinical sleep disturbance, which is mediated by hormone derangement, in particular reduced oestrogen, progesterone and melatonin levels [164]. Oestrogen is required for the metabolism of neurotransmitters (norepinephrine and serotonin) involved in regulating sleep patterns, and plays an important role in maintaining a low body temperature at night [165]. Progesterone has sedative and anxiolytic actions that can support sleep quality, and acts as a respiratory stimulant that lessens airway resistance in obstructive sleep apnoea (OSA) [166]. Melatonin is a neuroendocrine hormone that is widely recognised as crucial in maintaining circadian rhythm regulation. However, melatonin is also involved in ovarian function, with actions including delaying ovarian senescence, promoting follicle formation and improving oocyte quality [167–173].

The current PCOS guideline recognises that OSA is 6.5-8.3 times more likely in women with PCOS [164, 174–177], and promotes routine screening to identify and treat associated symptoms, such as snoring, excessive sleepiness and the potential for fatigue to worsen mood disorders [18]. Screening should include a simple questionnaire, such as the Berlin tool [178], and where appropriate women should be referred onto a specialist for further assessment and treatment [18]. The guidelines also highlight that treatment of OSA in PCOS should not be used to improve metabolic features. Since guideline inception evidence has emerged reporting weight, PCOS and sleep are interrelated factors that can each contribute to the worsening presentation of one another, whereby sleep disorders and disturbance may worsen the presentation of PCOS related metabolic outcomes and vice versa [179].

Hypersomnia and insomnia are also common clinical sleep disorders in PCOS [164, 177, 180], with prevalence estimated at 11% versus 1% in women with versus those without PCOS [180]. Even in the absence of clinically diagnosed sleep disorders, women with PCOS have a higher prevalence of sleep disturbances, including poor sleep quality [181], issues with sleep initiation [182], severe fatigue [140], restless sleep [140] and difficulty sleeping overnight [140]. The prevalence of sleep disturbances may be up to 20% higher in women with PCOS compared to women without PCOS [183]. Emerging research also suggests that social restrictions arising from the COVID-19 pandemic have worsened sleep disturbances in women with PCOS [177]. Findings from key studies of non-clinical sleep disturbance can be found in Table 4.

**Table 4:** Key observational studies that report non-clinical sleep disruption in polycystic ovary syndrome

Reference	Sample size	Sleep methodology used	Main findings
Moran et al. 2015 [182]	PCOS: *n*=87 Non-PCOS: *n*=637	Modified version of the Jenkins Sleep Questionnaire	Women with PCOS were twice as likely to experience sleep disturbance PCOS was associated with difficulty falling asleep and maintaining sleep
Mo et al 2019 [140]^a^	PCOS: *n*=484 Non-PCOS: *n*=6094	Sleep duration was self-reported on a work day and non-work day Sleep quality was self-reported through frequency questions about difficulty falling asleep, restless sleep, difficulty sleeping and severe tiredness	Women with/without PCOS had similar sleep duration Women with PCOS had higher prevalence of sleep disturbance, and this relationship maintained even after controlling for BMI, depression, income, marital status, occupation, education status and COB
Bennett et al. 2021 [183]^a^	PCOS: *n*=464 Non-PCOS: *n*=5603	Sleep duration was self-reported on a work day and non-work day Sleep quality was self-reported through frequency questions about difficulty falling asleep, restless sleep, difficulty sleeping and severe tiredness	Overall women with PCOS had greater adverse sleep symptoms and higher DGI However, subgroup analysis revealed PCOS was only associated with a higher DGI in women with adequate sleep There was no association between PCOS and DGI in women with poor sleep The higher DGI observed in women with PCOS may only be maintained in women who achieve adequate amounts of good quality sleep
Shreeve et al. 2013 [167]	PCOS: *n*=15 Non-PCOS: *n*=18	Actigraphy, PSQI and ESS	Women with PCOS had higher night time melatonin levels Women with PCOS had reduced sleep when compared to controls
Kutanaee et al. 2019 [181]	PCOS: *n*=201 Non-PCOS: *n*=199	PSQI	Women with PCOS had lower sleep quality and daytime function Women with PCOS were more likely to utilise medication to assist with sleep

*Abbreviations*: *BMI* Body mass index, *COB* Country of birth, *DGI* Dietary Guidelines Index, *ESS* Epworth Sleepiness Scale, *PCOS* Polycystic ovary syndrome, *PQSI* Pittsburgh Sleep Quality Index

^a^Mo et al. [140] and Bennett et al. [183] share the same cohort

In the general population short and disturbed sleep is consistently associated with excess weight [184], IR [185], T2DM [185] and CVD [186]. Similar relationships are observed in PCOS, where OSA and sleep disordered breathing exacerbates risk of IR and metabolic consequences of abnormal glucose tolerance [187, 188]. A cross-sectional study in adolescents with PCOS (*n*=103) reported those with sleep disordered breathing had significantly higher BMI Z-scores, and a higher prevalence of metabolic syndrome (METS) [188]. Similar metabolic consequences are seen in women with PCOS who suffer from non-clinical sleep disturbance [164]. Underlying mechanisms linking sleep disorders and disturbance with worsened metabolic outcomes include amplified sympathetic tone and oxidative stress [164], reduced adipose tissue lipolysis, and an increase in energy intake stemming from heightened hedonic and endocrine appetite signals [189].

Unfavourable effects on energy metabolism and appetite regulation, may explain why women with PCOS who display sleep disturbance have a reduced capacity to maintain dietary interventions [183]. Moreover, depression and anxiety share a bidirectional relationship with disrupted and reduced sleep [190], and as stated previously, interventions that improve mental health can help to increase engagement with dietary and physical activity recommendations [131]. Optimising sleep may therefore be an important consideration when promoting healthy lifestyle change in women with PCOS [183].

## Traditional, complementary and integrative medicine

The 2018 PCOS guideline includes recommendations on inositol supplementation, though do not include evidence regarding the use of other supplements, herbal medicine or other TCIM approaches, including acupuncture and yoga [18].

### Vitamins, vitamin-like supplements, minerals and other supplements

The 2018 guideline highlights that inositol (including myo-inositol (MI) and di-chiro inositol) is a nutritional supplement that may be involved in insulin signalling transduction [191]. MI in particular is a key endocrine regulator that displays impaired metabolism in PCOS [191]. MI supplementation has been explored in a meta-analysis of nine RCTs (*n*=496), which showed improved metabolic profiles and reduced hyperandrogenism [191]. These findings are supported by two earlier meta-analyses, reporting improved ovulation, menstrual cyclicity, and hormonal profiles following MI supplementation [192, 193]. The 2018 PCOS guideline recommends that inositol (in any form) should be considered as an experimental therapy in PCOS management. The guideline also recognises that women participating in any form of TCIM should be encouraged to advise their health professional. However, it does not consider emerging evidence for the use of other types of TCIM in PCOS treatment as this was outside of the scope of the 2018 guideline.

#### Vitamins

B-group vitamins (B_1_, B_6_ and B_12_), folic acid (B_9_) and vitamins D, E, and K are critical for several biological processes that can affect metabolic and reproductive features of PCOS. B-group vitamins work alongside folic acid (the synthetic form of folate) to regulate homocysteine (Hcy) via re-methylation of Hcy to methionine [194]. Hcy is an amino acid that confers an increased risk of CVD at high levels, and which is often deranged in women with PCOS [195], likely related to a higher prevalence of folate deficiency [196–198]. One RCT explored the use of B-group vitamins combined with folic acid in 60 women with PCOS, and reported a reduction in the Hcy increasing effect of metformin [198]. Folic acid alone has also been examined in two RCTs of women with PCOS (*n*=69 [199] and *n*=81 [200]), improving FINS, HOMA-IR, C-reactive protein, total antioxidant capacity (TAC) and glutathione with doses ≥ 5 mg/day when compared with placebo [199, 200]. Regarding vitamin D supplementation, three large-scale meta-analyses reported improvements in measures of IR (HOMA-IR [201, 202], FINS [201]), fasting glucose [201]), lipid profiles (LDL-C [201–203], TC [203] and TAG [203]) and androgens (T) [202], when compared with placebo. While vitamin E (or tocopherol) has various reported benefits on fertility outcomes in other populations [204], and has improved androgen profiles when co-supplemented with coenzyme Q10 (CoQ10) in women with PCOS [205], to date no RCTs have examined the use of vitamin E supplements alone in PCOS. Vitamin K also has limited available literature in PCOS, with only one RCT (*n*=84) demonstrating improvements in anthropometry, insulin and androgen profiles following supplementation (90 μg/day Menaquinone-7 for eight weeks), compared with placebo [206].

#### Vitamin-like supplements

Vitamin-like supplements including bioflavonoids, carnitine and alpha-lipoic acid (α-LA) have well-recognised antioxidant properties and play a role in fatty acid and glucose metabolism, providing possible metabolic benefits in PCOS [207]. Bioflavonoids consist of plant-derived polyphenolic compounds, some of which have been inversely associated with METS in women with PCOS [207]. In a pilot prospective study of 12 women with PCOS, 36 mg/day of the soy isoflavone genistein for six months improved lipid profiles but not anthropometry, IR, hormonal profiles or menstrual cyclicity [208]. Carnitine, particularly the active form L-carnitine, is reported to be lower in women with PCOS and linked with hyperandrogenism, hyperinsulinaemia and reduced oocyte quality [209, 210]. One RCT explored L-carnitine use in PCOS and found beneficial effects on mental health parameters and markers of oxidative stress [211], although the integrity of these have come under scrutiny and hence should be interpreted with caution [122,212]. Regarding α-LA, a small pre-post study (*n*=6) administered 1200 mg/day for 16 weeks, and reported improved IR, LDL-C and TAG, though no effects on TAC or plasma oxidation metabolites [213]. Another RCT reported improved anthropometric (BMI), metabolic (FINS and HDL-C) and reproductive (menstrual cyclicity) features in 46 women with PCOS receiving α-LA supplementation (600 mg/day for 180 days) compared with controls [214]. However, as these women were co-supplemented with 1000 mg/day D-chiro-inostiol, findings are not isolated to the effects of α-LA alone [214].

#### Minerals

Minerals such as calcium, zinc, selenium, magnesium and chromium picolinate (CrP) have been explored in PCOS due to their reported insulin sensitising, antioxidant and anti-inflammatory properties [215–217]. A small number of studies have also reported women with PCOS are at higher risk of being deficient in calcium [218], zinc [215, 217] and selenium [195]. A recent systematic review (six RCTs) reported that vitamin D and calcium co-supplementation in women with PCOS improved lipid and androgen profiles, follicular health and menstrual cyclicity [219]. While these findings are promising, it is difficult to attribute benefits to calcium alone, given calcium is often co-supplemented with vitamin D due to their complementary mechanisms of action. One systematic review (five RCTs) in PCOS reported zinc (often co-supplemented with other nutrients such as calcium, vitamin D and magnesium), improved HOMA-IR, lipids, T, FSH and DHEAS [220] compared to placebo. Another systematic review (five RCTs) examining selenium supplementation reported reduced IR, oxidative stress and inflammation, while results for anthropometry, lipids, androgens and hirsutism were inconsistent [221]. Regarding magnesium (an intracellular cation involved in insulin metabolism), while supplementation in PCOS has been associated with reduced IR in observational research [222], these findings are not supported by data from RCTs, with considerable inconsistencies between studies [222]. Two meta-analyses examined CrP in women with PCOS [223, 224]. While one reported that CrP supplementation reduced BMI, FINS and free testosterone [223], the other reported decreased IR, but not BMI, and increased levels of T [224].

#### Other supplements

Other supplements purported to provide a range of antioxidant and anti-inflammatory benefits, including omega-3 fatty acids, N-acetyl-cysteine (NAC), CoQ10, probiotics, quercetin, resveratrol and melatonin have been explored in PCOS. A meta-analysis (nine RCTs) of women with PCOS (*n*=591) receiving omega-3 supplementation reported reductions in HOMA-IR, TC, TAG and LDL-C, though showed no effect on other metabolic parameters or T [225]. In a meta-analysis of eight RCTs (*n*=910) examining NAC supplementation (the acylated form of L-cysteine), researchers reported improved glucose regulation and a greater likelihood of conception and livebirths in women with PCOS compared with placebo [226]. In a single RCT (*n*=60) CoQ10 supplementation (100 mg/day for 12 weeks) improved fasting glucose and insulin, HOMA-IR, insulin sensitivity index and TC, compared with the placebo group [227]. Two meta-analyses reported probiotics improved FAI, SHBG, IR and blood lipids, with no differences in weight or hirsutism between intervention and placebo groups [228, 229]. These findings may be linked to lower microbial diversity and increased intestinal permeability in women with PCOS [230, 231]. In regards to quercetin and resveratrol, which are both food derived polyphenols with a strong antioxidant capacity, one systematic review (three experimental studies, *n*=246 women with PCOS) reported quercetin supplementation improved measures of IR and testosterone levels, but not anthropometry compared with placebo [232]. Similarly, one RCT in women with PCOS (*n*=61) reported resveratrol (800-1500 mg/day for four days) improved androgen and metabolic profiles and oocyte and embryo quality compared with placebo [233]. Finally, a systematic review (two RCTs and one cell-culture study) investigating the effects of melatonin supplementation in women with PCOS using assisted reproductive technologies reported melatonin significantly increased clinical pregnancy rates but not live birth rates [172]. A more recent RCT (*n*=56) reported improved levels of T, hirsutism, inflammatory and oxidative stress profiles in women receiving 10 g melatonin/day for 12 weeks, compared with placebo [234].

### Herbal medicine

To date the most recent and comprehensive review (Cochrane review including five RCTs and *n*=414 women with PCOS) investigating the effects of herbal medicine on reproductive outcomes, reported no difference between the use of Chinese herbal medicine (CHM) and clomiphene for pregnancy rates, and limited evidence of increased pregnancy rate for CHM with clomiphene compared with clomiphene alone [235]. This review concluded that there was inadequate evidence to promote the use of CHM for the treatment of subfertility in women with PCOS [235]. Similarly, a smaller systematic review (five studies) investigating the effects of four herbal medicines (green tea, cinnamon, spearmint and black cohosh) on menstrual regularity in PCOS, found limited high-quality evidence from RCTs to support their clinical use and concluded that evidence for safety was lacking [236].

More recently, a number of small RCTs investigating metabolic and reproductive effects of a range of herbal medicines have been published. Curcumin, an active compound in turmeric (*Curcuma longa),* may exert hypoglycemic effects via a number of mechanisms, including attenuation of circulating levels of tumor necrosis factor-α [237]. One RCT (*n*=67) reported decreased levels of fasting glucose following supplementation compared with placebo [238], while another (*n*=51) which used a lower dose (1000 mg/day versus 1500 mg/day) and shorter duration (six weeks versus 12 weeks), reported no between group differences for fasting glucose, HOMA-IR or lipids [239]. *Salvia officinalis* or sage contains multiple active compounds that display antioxidant effects and therefore effects on glucose metabolism and insulin sensitivity [240]. One RCT (*n*=72) reported consuming sage extract for eight weeks improved IR and reduced BMI, with no effects on WHR or blood pressure [241] *Foeniculum vulgare* or fennel may provide protective effects on hormonal abnormalities in PCOS via its actions as a phytoestrogen [242]. One RCT (*n*=55) reported that six months of fennel tea and dry cupping was as effective as metformin for reducing BMI and menstrual cycle length [243]. *Glycyrrhiza glabra* or licorice contains active phytochemicals including isoflavane and glabridin, which have been shown to have antiandrogenic effects [244]. Two experimental studies in healthy women (*n*=9) [245] and women with PCOS (*n*=32) [246] reported that 3.5 g/day of licorice extract decreased T [245] and reduced side effects of spironolactone [246]. *Mentha spicata* (spearmint), *Zingiber offinale Roscoe* (ginger), *Cinnamomum cassia* (cinnamon) and *Citrus sinensis* (citrus) have been shown to exert anti-inflammatory and hypoglycemic effects [247–250]. One RCT in infertile women with PCOS (*n*=60) comparing the effects of a herbal mixture (citrus, ginger, cinnamon and spearmint) with clomiphene citrate (CC), herbal mixture alone, or CC alone reported that the herbal mixture, with or without CC, improved circulating antioxidant levels, IR and fasting blood glucose, but not menstrual regularity when compared to CC alone [251]. While observations from emerging research are promising, to support the safe translation of findings into the clinical setting there is a clear need for larger clinical trials investigating the efficacy and safety of herbal medicine use in PCOS.

### Other traditional, complimentary and integrative medicine approaches

Acupuncture may provide beneficial impacts on sympathetic function [252] and ovarian blood flow [253] in women with PCOS. A recent meta-analysis of 22 RCTs (*n*=2315 women with PCOS) reported recovery of the menstrual period in the acupuncture group when compared with placebo, but no evidence for differences between groups in terms of live birth, pregnancy and ovulation [254]. While an earlier meta-analysis reported a significant reduction in BMI following acupuncture use, this was mainly due to one RCT (*n*=80) which compared acupuncture and the oral contraceptive pill to the oral contraceptive pill alone [255]. When this study was removed, the pooled analysis was no longer significant [255].

Yoga gymnastics have been recommended as an example of moderate physical activity in the 2018 evidence-based PCOS guideline [18]. However, as yoga is considered a mind-body therapy that incorporates aspects of meditation, it may provide additional benefits beyond those gained through other forms of exercise [256]. While one systematic review (16 observational and experimental studies, *n*=365 women with PCOS) reported yoga may provide a range of psychological, reproductive and metabolic benefits, no meta-analysis was performed and a limited summary of included studies made it difficult to confirm findings [257]. A more recent systematic review (11 experimental studies) included a meta-analysis of two RCTs and found that yoga significantly decreased clinical hyperandrogenism, menstrual irregularity and fasting glucose and insulin [258]. Lastly, findings from a recent RCT (*n*=67 women with PCOS) suggests that 90 minutes of yoga per day for six weeks can significantly reduce hirsutism, waist and hip circumference when compared to controls [259]. Please see Table 5  for a summary of available evidence from meta-analyses and experimental studies investigating the effects of TCIM on PCOS outcomes.

**Table 5:** Reviews and experimental studies investigating the effects of traditional, complimentary and integrative medicine on polycystic ovary syndrome outcomes

Intervention	N study design	N studies; N participants	Main findings^a^	References
**Vitamins**				
B-group vitamins (B1, B6, and B12)	1 RCT	1; 60	Counteracted Hcy-increasing effect of metformin ↔ HOMA-IR	Kilicdag et al. 2005 [198]
Folate (vitamin B9)	2 RCT	2; 150	↓ Hcy [199, 200], HOMA-β [199], HOMA-IR [200], FINS [200], TC:HDL-C ratio [200], CRP [199], MDA [199] ↑ TAC [199], GSH [199]	Bahmani et al. 2014 [199] Asemi et al. 2014 [200]
Inositols (vitamin B8)	1 SR/MA	9 RCT; 496	↓ HOMA-IR; ↓ FINS ↔ androstenedione, T, SHBG	Unfer et al. 2017 [191]
Vitamin D	2 SR/MA	23 RCT; 1367	↓ TC [201], LDL [201], TAG [201], HOMA-IR [203], FGL [203], FINS [203], VLDL-C [203] ↑ QUICKI [203] ↔ HDL-C [201]	Guo et al. 2020 [201] Gao et al. 2021 [203]
Vitamin E	1 RCT	1; 86	↓ FGL, HOMA-IR, SHBG, T (only when combined with coenzyme Q10)	Izadi et al. 2019 [205]
Vitamin K	1 RCT	1; 79	↓ WC, FBM, FINS, HOMA-IR, HOMA-β, TAG, FAI, DHT ↑ skeletal muscle mass, SHBG, QUICKI	Tarkesh et al. 2020 [206]
**Vitamin-like supplements**				
Soy isoflavones	1 pilot pre-post prospective	1; 12	↓TC, LDL-C, LDL-C:HDL-C ratio, TAG	Romualdi et al. 2018 [208]
Carnitine (L-Carnitine)	1 RCT	1; 60	↓ MDA, MDA:TAC ratio ↑ TAC	Jamilian et al. 2017 [211]
Alpha-lipoic acid	2 pre-post prospective	2; 52	↓ BMI [214], IR [213]**,** LDL-C [213], TAG [213], ovarian cysts [214] ↑ progesterone [214]	Masharani et al. 2010 [213] Cianci et al. 2015 [214]
**Minerals**				
Vitamin D and calcium	1 SR/MA	6 RCT; 480	↓ FINS, HOMA-IR, FGL, T, TAG, VLDL-C, TC, LDL-C, hirsutism ↑ QUICKI, menstrual regularity	Shojaeian et al. 2019 [219]
Zinc	1 SR	5 RCT; 285	↓ HOMA-IR, HOMA-β, FINS, MDA, CRP, T, FSH, TC, LDL-C, TAG, VLDL-C, DHEAS ↑ TAC, QUICKI	Nasiadek et al. 2020 [220]
Selenium	1 SR	5 RCT; NR	↓ IR, CRP and MDA in some RCTs ↔ (or inconsistent findings) BMI, BW, FGL, blood lipids, androgens, acne, hirsutism	Hajizadeh-Sharafabad et al. 2019 [221]
Magnesium	1 SR	3 RCT; 156	Serum magnesium concentrations were associated with IR but supplementation had inconsistent effects	Hamilton et al. 2019 [222]
Chromium Picolinate	2 SR/MA	11 RCT; 702	↓ BMI [223], FINS [223], IR [224], T [223] ↑ T [224] ↔ BMI [224], FG [223]	Fazelian et al. [223] Tang et al. [224]
**Other supplements**				
Omega-3 fatty acids	1 SR/MA	9 RCT; 591	↓ HOMA-IR, TC, LDL-C and TAG. ↔ FINS, FGL, BMI, androgens	Yang et al. 2018 [225]
N-acetyl-cysteine	1 SR/MA	8 RCTS; 910	↑ rates of pregnancy and live births	Thakker et al. 2015 [226]
Coenzyme Q10	1 RCT	1; 60	↓FGL, FINS, HOMA-IR, HOMA-β, , TC, LDL-C ↑ QUICKI	Samimi et al. 2017 [227]
Probiotics	2 SR/MA	19 RCT; 1261	↓ FINS [228], TG [228], VLDL-C [228], FAI [229] ↑QUICKI [228], SHBG [229] ↔ BW [228], FGL [228], HOMA-IR [228], TC [228], LDL-C [228], HDL-C [228], CRP [228], DHEA [228], T [229]	Liao et al. 2018 [228] Shamasbi et al. 2020 [229]
Quercetin	1 SR	3 RCT; 246	Some improvement in adiponectin-mediated IR ↔ BW, WHR	Pourteymour et al. 2020 [232]
Resveratrol	1 SR/MA	3 RCT; 131	↓ T ↑ high-quality oocytes and embryos ↔ BMI, blood lipids, FGL, pregnancy rate	Shojaei-Zarghani et al. 2021 [233]
Melatonin	1 SR/MA	2 RCT and 1 cell culture; 640	↑ pregnancy rates in assisted reproductive technology	Hu et al. 2020 [172]
**Herbal medicine**				
Cinnamon	1 SR/MA	5 RCT; 448	↓HOMA-IR, TC, LDL, FGL, FINS ↑ HDL ↔ BW	Heydarpour et al. 2020 [260]
Curcumin	2 RCT	2; 118	↓ FGL [238], DHEA [238] ↔ FGL [239], FINS [238], blood lipids [239], IR [239]	Heshmati et al. 2021 [238] Sohaei et al. 2019 [239]
Sage	1 RCT	1; 70	↓ BW, BMI, WC, FGL, FINS, HOMA-IR, QUICKI ↔ WHR	Amini et al*.* 2020 [241]
Fennel and dry cupping	1 RCT	1; 55	↓ BMI, cycle length	Mokaberinejad et.al. 2019 [243]
Licorice	1 pre-post prospective 1 quasi-experimental	2; 41	↓ T [245] Reduce prevalence of side effects related to the diuretic activity of spironolactone [246]	Armanini et al. 2004 [245] Armanini et al. 2007 [246]
Spearmint, ginger, citrus and cinnamon	1 RCT	1; 60	↓ HOMA-IR, FINS, FGL	Ainehchi et al. 2019 [251]
Chinese herbal medicine	1 SR/MA	4 RCT; 414	↑ pregnancy rate when taken with clomiphene (versus clomiphene alone) ↔ pregnancy rate when taken alone (versus clomiphene alone) Insufficient evidence for subfertility	Zhou et al. 2016 [235]
**Other TCIM**				
Acupuncture	2 SR/MA	31 RCT; 2846^b^	↓ BMI [255], LH [254], T [254] ↑ menstrual regularity [254] ↔ FGL [255], FINS [255], live birth [254], pregnancy rate [254], ovulation [254]	Wu et al. 2020 [254] Qu et al. 2016 [255]
Yoga	2 SR [120, 257] 1 SR/MA [258] 1 RCT [259]	21; 1059 ^a^	↓ WC [259], HC [259], HOMA-IR [120], FGL [258], FINS [258], T [120], LH [120], DHEA [120], androstenedione [120], adiponectin [120], clinical hyperandrogenism [259] ↑ menstrual regularity [258], menstrual frequency [257] ↓ stress and anxiety [257]	Shele et al. 2020 [120] Thakur et al. 2021 [257] Anita et al. 2021 [258] Mohseni M et al. 2021 [259]

*Abbreviations:* ↑ significant increase (*P* ≤ 0.05), ↓ significant decrease (*P* ≤ 0.05), ↔ no significant change, *BMI* Body mass index, *BW* Body weight, *DHEAS* Dehydroepiandrosterone-sulfate, *DHT* Dihydrotestosterone, *FGL* Fasting glucose level, *FINS* Fasting insulin level, *FBM* Fat body mass, *FSH* Follicle stimulating hormone, *FT* Free testosterone, *GSH* Glutathione, *HC* Hip circumference, *Hcy* Homocysteine, *HOMA-IR* Homeostatic assessment of insulin resistance, *HDL-C* High density lipoprotein cholesterol, *IR* Insulin resistance, *QUICKI* Quantitative insulin sensitivity check index, *QoL* Quality of life, *MDA* Malondialdehyde, *MA* Meta-analysis, *NR* Not reported, *OCP* Oral Contraceptive Pill, *RCT* Randomised controlled trial, *SHBG* Sex hormone binding globulin, *SR* Systematic review, *T* Testosterone, *TAC* Total antioxidant capacity, *TC* Total cholesterol, TAG Triglycerides, *TCIM* Traditional, complimentary and integrative medicine, *VLDL-C* Very low density lipoprotein cholesterol, *WC* Waist circumference, *WHR* Wait hip ratio

^a^Summarises commonly used measures in PCOS research and does not report on all measured outcomes. For prospective pre-post studies significant changes from baseline are reported. For RCTs significant changes between intervention(s) and control and reported

^b^Not all participants are included in the findings reported here (e.g. where findings from subgroup analysis are reported)

## Summary of findings and research gaps

The 2018 International Evidence-Based Guideline for the Assessment and Management of PCOS highlights lifestyle (diet, physical activity and/or behavioural) management as the primary initial treatment strategy [18]. It is important to consider that the definition of lifestyle management may warrant expansion consistent with the whole person model of healthcare provision, which may include care addressing psychological and sleep interventions, as well as a range of TCIM approaches [20]. In line with patient interest [31–35], and to assist women and healthcare providers in understanding the evidence to aid safe implementation of adjunct therapies, rigorous assessment of the evidence for these alternative lifestyle strategies in PCOS management in warranted. Using a holistic definition of patient care, this review has summarised evidence to date on the traditional components of lifestyle change (diet, physical activity and behavioural change), psychological interventions and non-pharmacological strategies (sleep, supplements, herbal medicine and other TCIM approaches). Table 6 provides a overview of current guideline recommendations alongside the key findings from this review, summarising the identified research gaps that need to be addressed before evidence-based recommendations for clinical practice can be updated.

**Table 6:** Current recommendations for clinical practice and research gaps identified by this review

Recommendation(s) from current guidelines^a^	Category of recommendation^b^	Research gaps
**Effectiveness of lifestyle interventions**		
Healthy lifestyle behaviours encompassing healthy eating and regular physical activity should be recommended in all those with PCOS to achieve and/or maintain healthy weight and to optimise hormonal outcomes, general health, and QoL across the life course.	CCR	• Improves sustainability of weight loss interventions. • Identifies subgroups who respond to weight loss with clinically relevant metabolic and reproductive improvements (this requires the inclusion of more clinical reproductive outcomes in RCTs). • Defines weight loss thresholds for improvements in different PCOS features (metabolic, reproductive and psychological). • Characterises the degree of metabolic and reproductive improvements related to different lifestyle factors (diet, physical activity and behavioural) independent of weight changes. • Considers effects of weight gain prevention on limiting the progression/worsening of PCOS features. • Investigates how different dietary, physical activity and behavioural interventions affect engagement, adherence and sustainability of lifestyle change. • Investigates efficacy and effectiveness of healthy lifestyle changes independent of weight change.
Lifestyle intervention (preferably multicomponent including diet, exercise and behavioural strategies) should be recommended in all those with PCOS and excess weight, for reductions in weight, central obesity and IR.	EBR	
Achievable goals such as 5% to 10% weight loss in those with excess weight yields significant clinical improvements and is considered successful weight reduction within six months. Ongoing assessment and monitoring is important during weight loss and maintenance in all women with PCOS.	CPP	
SMART (Specific Measurable, Achievable, Realistic and Timely) goal setting and self-monitoring can enable achievement of realistic lifestyle goals.	CPP	
Psychological factors such as anxiety and depressive symptoms, body image concerns and disordered eating, need consideration and management to optimise engagement and adherence to lifestyle interventions.	CPP	
Health professional interactions around healthy lifestyle, including diet and exercise, need to be respectful, patient-centred and to value women’s individualised healthy lifestyle preferences and cultural, socioeconomic and ethnic differences. Health professionals need to also consider personal sensitivities, marginalisation and potential weight-related stigma.	CPP	
Healthy lifestyle may contribute to health and QoL benefits in the absence of weight loss.	CPP	
Healthy lifestyle and optimal weight management appears equally effective in PCOS as in the general population and is the joint responsibility of all health professionals, partnering with women with PCOS. Where complex issues arise, referral to suitably trained allied health professionals needs to be considered.	CPP	
**Dietary interventions**		
A variety of balanced dietary approaches could be recommended to reduce dietary energy intake and induce weight loss in women with PCOS and overweight and obesity, as per general population recommendations.	CCR	• Low GI/GL diets may provide benefits in reducing weight and IR in women with PCOS. Further research needs to assess additional risk factors including reproductive function and CVD risk. • Identify and define the optimal diet for PCOS management by comparing a range of different dietary approaches (e.g. DASH, Mediterranean or low GI/GL).
General healthy eating principles should be followed for all women with PCOS across the life course, as per general population recommendations.	CCR	
To achieve weight loss in those with excess weight, an energy deficit of 30% or 500 - 750 kcal/day (1,200 to 1,500 kcal/day) could be prescribed for women, also considering individual energy requirements, body weight and physical activity levels.	CPP	
In women with PCOS, there is no or limited evidence that any specific energy equivalent diet type is better than another, or that there is any differential response to weight management intervention, compared to women without PCOS.	CPP	
Tailoring of dietary changes to food preferences, allowing for a flexible and individual approach to reducing energy intake and avoiding unduly restrictive and nutritionally unbalanced diets, are important, as per general population recommendations.	CPP	
**Physical activity interventions**		
Health professionals should encourage and advise the following for prevention of weight gain and maintenance of health: • in adults from 18 – 64 years, a minimum of 150 min/week of moderate intensity physical activity or 75 min/week of vigorous intensities or an equivalent combination of both, including muscle strengthening activities on 2 non-consecutive days/week; • in adolescents, at least 60 minutes of moderate to vigorous intensity physical activity/day, including those that strengthen muscle and bone at least 3 times weekly; • activity be performed in at least 10-minute bouts or around 1000 steps, aiming to achieve at least 30 minutes daily on most days.	CCR	While evidence supports the provision of supervised vigorous aerobic exercise, which may provide greater benefits on PCOS symptoms than other types of exercise (e.g. resistance training), additional larger and longer-term studies are required to: • Characterise optimal exercise prescription for PCOS management. • Identify factors that improve adherence to exercise interventions. • Identify subgroups who respond to exercise with clinical improvements.
Health professionals should encourage and advise the following for modest weight-loss, prevention of weight-regain and greater health benefits: • a minimum of 250 min/week of moderate intensity activities or 150 min/week of vigorous intensity or an equivalent combination of both, and muscle strengthening activities involving major muscle groups on 2 non-consecutive days/week; • minimised sedentary, screen or sitting time.	CCR	
Physical activity includes leisure time physical activity, transportation such as walking or cycling, occupational work, household chores, games, sports or planned exercise, in the context of daily, family and community activities. Daily, 10000 steps is ideal, including activities of daily living and 30 minutes of structured physical activity or around 3000 steps. Structuring of recommended activities need to consider women’s and family routines as well as cultural preferences.	CPP	
**Behavioural interventions**		
Lifestyle interventions could include behavioural strategies such as goal-setting, self-monitoring, stimulus control, problem solving, assertiveness training, slower eating, reinforcing changes and relapse prevention, to optimise weight management, healthy lifestyle and emotional wellbeing in women with PCOS.	CCR	• To identify behavioural and cognitive strategies that should be targeted in women with PCOS, more observational research that characterises women’s use of self-management strategies is needed. • To aid replication and interpretation of findings, RCTs must clearly define the theoretical frameworks and behavioural components used in intervention design.
Comprehensive health behavioural or cognitive behavioural interventions could be considered to increase support, engagement, retention, adherence and maintenance of healthy lifestyle and improve health outcomes in women with PCOS.	CPP	
**Assessment and treatment of infertility (as it relates to alcohol and smoking use)** **Cardiovascular disease risk (as it relates to alcohol and smoking use)**		
Factors such as blood glucose, weight, blood pressure, smoking, alcohol, diet, exercise, sleep and mental, emotional and sexual health need to be optimised in women with PCOS, to improve reproductive and obstetric outcomes, aligned with recommendations in the general population.	CPP	• Determine whether women with PCOS are at a higher risk of alcohol and smoking-related infertility complications (with a focus on anovulatory infertility) when compared to women without PCOS. • Determine whether women with PCOS are at a higher risk of smoking-related CVD complications when compared to women without PCOS.
If screening reveals CVD risk factors including obesity, cigarette smoking, dyslipidemia, hypertension, impaired glucose tolerance and lack of physical activity, women with PCOS should be considered at increased risk of CVD.	CCR	
**Quality of life**		
Health professionals and women should be aware of the adverse impact of PCOS on quality of life.	CCR	• Validate QoL tools longitudinally to identify clinically meaningful differences in QoL scores.
Health professionals should capture and consider perceptions of symptoms, impact on quality of life and personal priorities for care to improve patient outcomes.	CCR	
The PCOS quality of life tool (PCOSQ), or the modified PCOSQ, may be useful clinically to highlight PCOS features causing greatest distress, and to evaluate treatment outcomes on women’s subjective PCOS health concerns.	CPP	
**Depression and anxiety symptoms, screening and treatment** **Psychosexual function** **Body image** **Eating disorders and disordered eating**		
Health professionals should be aware that in PCOS, there is a high prevalence of moderate to severe anxiety and depressive symptoms in adults; and a likely increased prevalence in adolescents.	CCR	• To determine accurate prevalence of psychological conditions in PCOS, more adequately powdered cross-sectional studies using structured diagnostic interviews administered by appropriately qualified professionals are required. • Future research should consider the efficacy of different types of psychological interventions in PCOS, with a focus on how changes to mental health symptoms influence engagement with lifestyle change. In particular, the development of a PCOS specific CBT program, tailored to meet the specific mental health needs of women with PCOS is warrant.
Anxiety and depressive symptoms should be routinely screened in all adolescents and women with PCOS at diagnosis. If the screen for these symptoms and/or other aspects of emotional wellbeing is positive, further assessment and/or referral for assessment and treatment should be completed by suitably qualified health professionals, informed by regional guidelines.	CCR	
If treatment is warranted, psychological therapy and/or pharmacological treatment should be offered in PCOS, informed by regional clinical practice guidelines.	CCR	
Factors including obesity, infertility, hirsutism need consideration along with use of hormonal medications in PCOS, as they may independently exacerbate depressive and anxiety symptoms and other aspects of emotional wellbeing.	CPP	
All health professionals should be aware of the increased prevalence of psychosexual dysfunction and should consider exploring how features of PCOS, including hirsutism and body image, impact on sex life and relationships in PCOS.	CCR	
If psychosexual dysfunction is suspected, tools such as the Female Sexual Function Index can be considered.	CCR	
Health professionals and women should be aware that features of PCOS can impact on body image.	CCR	
All health professionals and women should be aware of the increased prevalence of eating disorders and disordered eating associated with PCOS.	CCR	
If eating disorders and disordered eating are suspected, further assessment, referral and treatment, including psychological therapy, could be offered by appropriately trained health professionals, informed by regional clinical practice guidelines.	CCR	
**Obstructive sleep apnoea (OSA)**		
Screening should only be considered for OSA in PCOS to identify and alleviate related symptoms, such as snoring, waking unrefreshed from sleep, daytime sleepiness, and the potential for fatigue to contribute to mood disorders. Screening should not be considered with the intention of improving cardiometabolic risk, with inadequate evidence for metabolic benefits of OSA treatment in PCOS and in general populations.	CCR	• To determine accurate prevalence of subclinical sleep disturbances in PCOS, more adequately powdered cross-sectional studies using validated subjective and objective sleep measures are required. • While emerging evidence suggests that disturbed sleep may exacerbate IR via decreasing energy expenditure and increasing adipose tissue deposition, more research in women with PCOS is needed to confirm this hypothesis. • Investigate effects of CBT interventions in women with PCOS who have disturbed sleep (outcomes of interest include food intake, metabolic rate, appetite hormones, weight, adherence to lifestyle changes and PCOS features).
A simple screening questionnaire, preferably the Berlin tool [178], could be applied and if positive, referral to a specialist considered.	CCR	
A positive screen raises the likelihood of OSA, however it does not quantify symptom burden and alone does not justify treatment. If women with PCOS have OSA symptoms and a positive screen, consideration can be given to be referral to a specialist centre for further evaluation.	CPP	
**Inositol**		
Inositol (in any form) should currently be considered an experimental therapy in PCOS, with emerging evidence on efficacy highlighting the need for further research.	EBR	• To reduce heterogeneity across studies investigating supplements or herbal medicine, RCTs should focus on specific populations within PCOS (i.e. age, BMI or phenotype) and adopt more consistent approaches to formulation (i.e. limit co-supplementation), dosage, intervention duration and the type of comparator used. • Mechanistic studies are needed to investigate herb- or nutrient-drug interactions (with common pharmacological treatments used in PCOS) and other possible interactions with the biological processes underpinning PCOS. • Research that characterises the uptake of TCIM approaches by women with PCOS, including where they are sourcing information on this topic, will aid health professionals understanding of how to safely navigate the use of adjunct therapies in PCOS management.
Women taking inositol and other complementary therapies are encouraged to advise their health professional.	CPP	

*Abbreviations*: *BMI* Body mass index, *CBT* Cognitive behavioural therapy, *CVD* Cardiovascular disease, *DASH* Dietary approaches to stop hypertension, *GI* Glycaemic index, *GL* Glycaemic load, *IR* Insulin resistance, *NAC* N-acetyl-cysteine, *PCOS* Polycystic ovary syndrome, *RCT* Randomised controlled trial, *QoL* Quality of life

^a^Recommendations are taken directly from the 2018 International Evidence-Based Guideline for the Assessment and Management of PCOS [18]. Does not include all recommendations, only those relevant to the findings of this review are presented

^b^*EBR* Evidence based recommendations: Evidence sufficient to inform a recommendation made by the guideline development group. *CCR* Clinical Consensus Recommendations: In the absence of evidence, a clinical consensus recommendation has been made by the guideline development group. *CPP* Clinical Practice Points: Evidence not sought. A practice point has been made by the guideline development group where important issues arose from discussion of evidence-based or clinical consensus recommendations

With regards to traditional lifestyle treatment, the majority of studies focussed on weight loss as a primary treatment goal. This indicates more research is warranted to understand the role of diet and exercise in lean women and/or in weight gain prevention. RCTs using lifestyle interventions under isocaloric conditions that investigate effects on IR, body composition and androgens independent of weight loss are needed. Given the high risk of failure with long-term weight management [9, 37, 40, 261] and high attrition in weight loss trials in PCOS [13], exploring interventions that focus on weight neural messaging around dietary quality and physical activity may also aid in optimising engagement, adherence and sustainability of lifestyle interventions. Future research should also identify subgroups who respond more favourably to weight loss [45, 53], to aid provision of a more targeted and personalised treatment approach.

With regards to diet strategies, there is a need for more research understanding the impact of low GI/GL diets on androgen status, as well as the biological mechanisms by which low GI/GL diets may impact reproductive and cardiometabolic outcomes associated with PCOS. With regards to physical activity, additional longer-term studies are required to guide exercise prescription in PCOS, although promising evidence supports the provision of vigorous aerobic exercise performed under supervised conditions (i.e. through referral to an exercise physiologist). While behavioural interventions are essential for long term sustainability of dietary and physical activity change, research in PCOS is scarce and interventions are not well defined. Future research should incorporate appropriate theoretical frameworks and clearly outline behavioural components utilised. This will aid intervention duplication and tailoring of active elements to ensure relevance in women with PCOS.

There is currently a lack of research investigating whether women with PCOS are at a higher risk of alcohol and smoking-related complications. This is particularly relevant given the well-established relationship between higher alcohol and cigarette use and rates of depression and anxiety in the general population [262–265]. There is also a need to better understand the relationship between alcohol intake and reproductive outcomes (particularly anovulatory infertility) [139], as safe alcohol limits in PCOS is currently unknown [139].

With regards to psychological interventions, the current evidence base for prevalence of mental health concerns in PCOS relies heavily on symptom prevalence. More adequately powered, gold standard prevalence studies using structured diagnostic interviews administered by appropriately qualified professionals are needed. While QoL has recently been highlighted as a core outcome in PCOS research [266], the application of QoL tools in clinical care is still unclear, with research yet to validate QoL tools longitudinally or identify clinically meaningful differences in QoL scores. The emerging evidence showing support for the use of CBT in PCOS [152–154] highlights an opportunity for tailoring of this psychological intervention to meet the specific mental health needs of women with PCOS, with a focus on how management of mental health symptoms affect lifestyle modifications. CBT that incorporates elements of mindfulness-based stress reduction also warrants further investigation.

Future research in PCOS and sleep disorders should include more high-quality research in subclinical disorders using objective sleep measures (polysomnography and actigraphy). Future work should also consider emerging evidence showing that disturbed sleep can detrimentally effect energy expenditure, which may increase adipose tissue deposition and exacerbate IR [164, 184, 186, 267–271], thereby worsening the presentation of PCOS. Further, a consideration of how sleep disturbance can reduce engagement with positive lifestyle changes, for example through the disruption of appetite regulation [272, 273] or via contributing to poor mental health outcomes [190, 274], is warranted. CBT interventions including elements of stimulus control and psychoeducation are effective non-pharmacological treatments for both clinical sleep disorders and sleep disturbances in the general population [275–277]. RCTs in women with PCOS that investigate effects of CBT on dietary intake, energy metabolism, appetite regulation, anthropometry, adherence to lifestyle changes and PCOS features are required.

With regards to TCIM, there is a vast array of literature suggesting some beneficial effects of vitamins (B-group vitamins, folate, vitamins D, E and K), vitamin-like nutrients (bioflavonoids, carnitine and α-LA), minerals (calcium, zinc, selenium, and CrP) and other formulations (such as melatonin, omega-3 fatty acids, probiotics, NAC and cinnamon) in PCOS [278]. However, the quality of evidence across studies ranges from meta-analyses of RCTs (vitamin D, omega-3 fatty acids and NAC) to single retrospective observational studies (vitamin K and carnitine). In addition, heterogeneity in results related to factors including variable PCOS presentation and study methodology make it difficult to draw definite conclusions. Future research should focus on specific populations within PCOS, for example age, BMI or phenotype (factors which substantially affect nutrient sufficiency), and outline more consistent approaches to supplement formulation, dosage, intervention duration and type of comparator used. Mechanistic studies are also needed to investigate herb- or nutrient-drug interactions (with common pharmacological treatments used in PCOS) and other possible interactions with the biological processes underpinning PCOS. In regards to acupuncture and yoga, more sufficiently powered RCTs are needed to determine clinical relevance and integration into PCOS management is not yet warranted.

While current research is not sufficiently robust to support integration of TCIM into routine clinical practice, healthcare providers should broaden their knowledge pertaining to how these therapies can be safely and appropriately utilised as adjuncts to conventional medical management [279–281]. TCIM is frequently used by women, with uptake of TCIM approaches increasing steadily over the past 10 years [31–35]. In women with PCOS, one cross-sectional study (*n*=493) found that 70% reported use of TCIM, namely nutritional and herbal supplements [282]. The most common reasons for use were to treat PCOS symptoms, improve general wellbeing and reduce depression. Of the women using TCIM, 77% had consulted with a complementary practitioner (acupuncturists, chiropractors, naturopaths and massage therapists) [282]. While the study did not report participants engagement with medical physicians, research in the general population has shown that patients are resistant to discuss TCIM use with their consulting physician [283–288]. Qualified healthcare providers should be involved in TCIM discussions to help ensure appropriate use, maximise possible benefits and minimize potential harm [289]. For example, to sustain patient engagement in women who express the desire to experiment with supplementation, healthcare providers could consider inositol supplementation, using a nuanced and case-specific approach that encapsulates the variety of pathologies in PCOS.

When considering all of the research summarised here, across traditional lifestyle, psychological, sleep and TCIM interventions, there is a clear need for more real-world PCOS research. This involves the translation of findings from clinical trials (where highly selected populations, intensive treatment protocols and expert multidisciplinary teams provide an ideal research setting), into the heterogenous situations that face clinicians [290–292]. Health professionals provide care to women from diverse social contexts, are often restrained by finite resources and are required to juggle many competing demands for their time [290–292]. While some barriers to implementation, including time, resource and access issues are considered in the current PCOS guideline, they were generated by the guideline development groups and research is needed to validate and clarify their proposed concerns. Real-world research is required to: a) fully understand whether lifestyle recommendations can be practically integrated into current healthcare settings; b) tailor interventions to meet the unique needs of women with PCOS; and c) generate evidence on clinical outcomes that are of great relevance to patients and clinicians, such as live birth, miscarriage and menstrual regularity, which can be collected through routine care.

It is also important to highlight that while lifestyle management is a first-line treatment for PCOS, the addition of pharmacological therapies to further improve clinical features of hyperandrogenism, menstrual irregularity and infertility are often indicated [293]. In these instances, prescribing physicians should consider how medical management and lifestyle change can be used in adjunct to optimise treatment. For example, the use of combined oral contraceptive pills may have detrimental effects on weight gain [294] and mental health [295], which can be mitigated by appropriate lifestyle intervention. Further, the combination of lifestyle modification and metformin has been shown to lower BMI, subcutaneous adipose tissue and improve menstruation compared with lifestyle modification alone, and hence may have an additive effect on improving cardio-metabolic outcomes in high risk groups [296].

## Conclusion

Using the whole person or holistic definition of health, this review has highlighted emerging areas of research that could be considered for integration into future classifications of lifestyle management in PCOS. When developing lifestyle recommendations for PCOS management, interpreting and communicating evidence not only for diet, physical activity and behavioural interventions, but also psychological, sleep and TCIM approaches, will aid clinicians to deliver patient-centred care by affording women more choice and therefore autonomy over their treatment options. This sentiment aligns with the core objectives underpinning the 2018 PCOS guideline, which sought to understand the unmet needs of women with PCOS through continuing to engage consumers in co-design of guideline development, implementation, translation and dissemination.

## Data Availability

Not applicable.

## Abbreviations

α-LA: Alpha-lipoic acid
BMI: Body mass index
CVD: Cardiovascular disease
CHM: Chinese herbal medicine
CrP: Chromium picolinate
CoQ10: Coenzyme Q10
CBT: Cognitive behavioural therapy
DASH: Dietary Approaches to Stop Hypertension
FINS: Fasting insulin level
FSH: Follicle stimulating hormone
FAI: Free androgen index
GI: Glycaemic index
GL: Glycaemic load
HDL-C: High density lipoprotein cholesterol
Hcy: Homocysteine
IR: Insulin resistance
LDL-C: Low density lipoprotein cholesterol
LH: Luteinizing hormone
VO_2max_: Maximal rate of oxygen
METS: Metabolic syndrome
MUFA: Monounsaturated fatty acid
MI: Myo-inositol
NAC: N-acetyl-cysteine
OGTT: Oral glucose tolerance test
PCOS: Polycystic ovary syndrome
PUFA: Polyunsaturated fatty acid
RCT: Randomised controlled trial
SHBG: Sex hormone-binding globulin
TAC: Total antioxidant capacity
TC: Total cholesterol
T: Testosterone
TCIM: Traditional, complementary and integrative medicine
TAG: Triglycerides
T2DM: Type 2 diabetes
WC: Waist circumference
WHR: Waist-hip-ratio
